# Mental health and mindfulness amongst Australian fire fighters

**DOI:** 10.1186/s40359-019-0311-2

**Published:** 2019-06-14

**Authors:** Isabelle Counson, Dominic Hosemans, Tara J. Lal, Brendan Mott, Samuel B. Harvey, Sadhbh Joyce

**Affiliations:** 1 Black Dog Institute, Sydney, NSW Australia; 2 School of Psychiatry, University of New South Wales, Sydney, NSW Australia; 3 Faculty of Medicine, Nursing and Health sciences, Monash University, Clayton, VIC Australia; 4 Fire and Rescue New South Wales, Sydney, Australia; 5 School of Health, University of New England, Parramatta, NSW Australia

**Keywords:** Mindfulness, Mental health, Fire fighters, Anxiety, Well-being, Depression

## Abstract

**Background:**

While extensive research has highlighted the positive mental health outcomes associated with mindfulness, little work has examined how mindfulness may protect the mental health of first responders exposed to trauma. This is important as there is increasing evidence that mindfulness skills, if protective, can be taught to groups of at-risk workers. The purpose of the current research was to examine the potential role mindfulness may have in supporting the mental health of Australian fire fighters.

**Methods:**

The sample consisted of 114 professional fire fighters who completed demographic and job-related questions followed by measures of mindfulness (FMI-14), well-being (WHO-5), depression (HADS-D) and anxiety (HADS-A). Hierarchical multiple linear regressions were performed to determine whether levels of mindfulness were associated with anxiety, depression and wellbeing after accounting for age and number of years of fire service.

**Results:**

High levels of mindfulness were associated with decreased depression (*p* ≤ .001) and anxiety (*p* ≤ .001) as well as increased psychological well-being (*p* ≤ .001). Measures of mindfulness were able to explain a substantial amount of the variability in well-being (26.8%), anxiety (23.6%) and depression (22.4%), regardless of age and years of fire service.

**Conclusions:**

The present study provides evidence for robust associations between dispositional mindfulness and mental health markers of depression, anxiety and well-being in Australian fire fighters recently exposed to trauma. Mindfulness is a psychological characteristic that may be able to be modified, although further research is required to substantiate these findings and to formally test mindfulness interventions. Such studies would allow greater insight into the underlying mechanisms through which mindfulness may exert its beneficial effects.

**Electronic supplementary material:**

The online version of this article (10.1186/s40359-019-0311-2) contains supplementary material, which is available to authorized users.

## Background

As part of their professional activities, first responders are regularly exposed to a wide range of physically and psychologically demanding stressors [1]. First responders, including police officers, fire fighters and ambulance personnel, intervene to assist and protect the community in emergency and crisis situations. There is a growing body of research that indicates that this type of emergency service work may come at a cost in terms of the mental health and wellbeing of those undertaking these vital activities [2]. Indeed, this specific population has been found to be at increased risk for developing psychological disorders [3]. The potential impact of poor mental health in emergency services occurs at a personal level, but also at an organisational and social level as many may be left unfit to work due to their distress, often requiring compensation and ongoing financial and medical support [2].

Most studies investigating the mental health of first responders have focused on post-traumatic stress disorder (PTSD) [2]. Berger et al. found in a large meta-analysis that one in ten first responders may be currently suffering from PTSD [4]. This is a rate considerably higher than those observed in the general population, where estimates for PTSD have been reported as 1.3% [5]. However, the potential impact of exposure to trauma among first responders may not be limited to PTSD alone [6]. Indeed, PTSD has been found to be highly co-morbid with other mental health conditions such as depression, general anxiety and substance abuse [5]. In addition, trauma exposure in emergency services is associated with an increased risk for major depression, anxiety [6] and problematic substance abuse independent of PTSD [7, 8].

Despite their frequent exposure to potentially traumatic events, only a minority of first responders appear to develop psychopathological symptoms [9]. While a general model of risk factors for developing psychopathology following trauma exposure is still lacking, a number of factors differentiating traumatised first responders from their non-traumatised colleagues have been identified [10]. Key risk factors may notably include age, years of service within emergency services, trauma intensity, coping styles as well as a history of personal trauma [3, 9]. Amongst these, there is particular interest in different coping mechanisms as some of these may be able to be taught or facilitated in order to enhance first responders resilience to trauma exposure [11].

Mindfulness can be defined as sustained attention and awareness of present moment experience in which each perceptible mental state and process is observed with a non-judgmental and accepting attitude [12]. While mindfulness is a psychological characteristic that varies as a trait amongst individuals, there has also been a suggestion that mindfulness skills can be improved with training [13]. Adaptive stress processing may be a key underlying mechanism through which mindfulness could enhance mental health and psychological well-being [14]. This line of research is consistent with a correlational study by Weinstein, Brown, and Ryan, who suggested that the buffering effect of mindfulness on depression may be more pronounced in participants exposed to high levels of stress compared to their peers experiencing lower levels of stress [15]. Two potential mechanisms may account for this. Firstly, it has been proposed that the attentional aspect of mindfulness may increase awareness of internal states and symptoms of stress thereby facilitating mindful individuals to respond promptly and effectively to stressful or threatening situations. Alternatively, an open and accepting attitude may promote a less defensive and avoidant response to potentially stressful or traumatic events [15]. According to Follette, Palm, and Pearson, mindfulness skills could increase willingness and aptitude to tolerate and process trauma-related emotions and cognitions without resorting to avoidant strategies [16]. In the wake of trauma and stress, the attentional broadening afforded by mindfulness may increase the ability to conduct a more accurate and informed assessment of the situations and potential options, thereby leading to a safe and effective response [17].

Similarly, the stress buffering hypothesis posits that mindfulness could mitigate stress appraisals and decrease stress-reactivity which would in turn promote the use of adaptive stress coping strategies [18]. Correlational studies have supported the notion that mindfulness could positively impact stress regulation processes by showing that mindfulness was related to both more benign stress appraisals and higher use of adaptive coping [15]. In line with these results, mindfulness was found to buffer against uncontrollable ruminative thinking that could prolong or aggravate depressed mood [19].

Despite a growing body of evidence documenting the mental health benefits of mindfulness, there has been little research investigating mindfulness in the context of emergency service work [20]. To our knowledge, only two studies have explored the relationships between mindfulness and mental health in populations of fire fighters.

A study conducted by Smith et al. investigated the associations between dispositional mindfulness, mental health and various measures related to work and stress exposure as well as variables assessing potential psychological resources such as social support [20]. The sample consisted of 124 urban fire fighters based in New Mexico. Hierarchical multiple regressions revealed that increased mindfulness was independently associated with decreased levels of PTSD symptoms, depressive symptoms, physical symptoms and alcohol misuse. While cross-sectional and precluding any conclusions regarding causation or direction, these preliminary findings highlighted mindfulness as a potentially important indicator of the mental health of fire fighters [20]. A more recent study conducted with 176 Italian fire fighters suggested that dispositional mindfulness could be a significant psychological resource for fire fighters’ mental health [21]. Stepwise multiple linear regression analyses indicated that low level of dispositional mindfulness in fire fighters was correlated with increased vicarious traumatisation as well as higher levels psychosomatic symptoms such as general dysphoria, social dysfunction and loss of confidence. In addition, mindfulness was more strongly associated with post-traumatic dimensions of arousal and intrusion than with other psychological variables such as loss of confidence or social dysfunction. The regression models examined in the study included various demographic indicators including age and seniority. However, there were no measures accounting for fire fighters’ levels of exposure to stress and trauma, which limited the strength of this study.

While promising, both of these studies can only offer preliminary insights into potential relationships between mindfulness and mental health in fire fighters. Further investigations should be conducted in various populations of emergency professionals and in different settings and geographical locations to replicate these initial results. The present study sought to investigate the relationships between various indicators of mental health and mindfulness in a sample of Australian fire fighters exposed to at least one traumatic event involving death or serious injury over the last 6 months. It was hypothesised that higher levels of mindfulness would predict decreased symptoms of (a) anxiety and (b) depression as well as increased (c) well-being, controlling for age and years worked with Fire and Rescue New South Wales (FRNSW).

## Method

### Participants

Fire and Rescue New South Wales (FRNSW), one of the world’s largest urban fire and rescue service granted approval for the present research to be conducted within 24 rescue stations across Sydney and surrounding regional areas such as Liverpool and Newcastle. Convenience sampling was used to recruit participants from the selected stations. The sample consisted of 143 professional fire fighters drawn from the selected stations. In line with the known demographics of this industry, the vast majority of participants were men, specifically 137 were male and six were female. Age ranged from 24 to 59 years (*M* = 42.33, *SD* = 8.70). Eligibility for participation in the study included the following criteria: (a) being currently employed as a permanent fire fighter within FRNSW; (b) being based at a fire and rescue station in New South Wales; (c) having good English comprehension; and (d) being above 18 years of age. In addition, given the specific aims of this study, participants had to have experienced trauma exposure over the last 6 months. This was measured via a question included in the survey that asked fire fighters to self-report the frequency of potentially traumatic events experienced over the last 6 months. Out of the 143 fire fighters recruited, a total of 114 (79.7%) fire fighters had been exposed to at least one traumatic event over the last 6 months. There were 109 males and five females with age ranged from 24 to 59 (*M* = 42.13, *SD* = 8.83). The highest proportion of fire fighters had worked over 20 years in FRNSW (*n* = 33; 28,9%).

### Materials

#### Demographics

Socio-demographic and occupational data included age, gender, educational level, years of experience within emergency services and more particularly within FRNSW.

#### Mindfulness

The short version of the Freiburg Mindfulness Inventory (FMI-14; [12]) was administered to assess participants’ levels of mindfulness, and more particularly their capacity to focus on the present moment in a non-evaluative manner. While the FMI-14 was developed based on Buddhist psychology, it was designed to be applicable to all populations including individuals without previous meditation experience. The scale comprises 14 self-report items rated on a four-point Likert-type scale from 1 (“rarely”) to 4 (“almost always”). A total score is calculated by summing the scores for the 14 items with higher scores reflecting higher levels of mindfulness. Previous research showed that the single-dimensional FMI-14 demonstrated stable and robust psychometric properties with good internal consistency (Cronbach’s alpha = 0.86; [12]). In addition, it was also found that the pattern of correlations obtained between FMI-14 scores and scores for measures of relevant constructs such as dissociation and meditation experience lent support to the construct validity of the FMI-14 [12]. The scale has been validated in different countries, where it has been shown to have similar reliability and validity as those found in the original study [22, 23].

#### Anxiety and depression

The Hospital Anxiety and Depression Scale (HADS; [24]) was created to detect states of anxiety and depression in non-psychiatric hospital clinics. The aim of the developers was to construct a reliable instrument that would carefully distinguish between depression and anxiety while avoiding any confounding effect from somatic disorders such as insomnia. The HADS is constituted of two seven-item subscales for anxiety (HADS-A) and depression (HADS-D). While the HADS-A items relate to the psychic manifestations of anxiety neurosis (e.g., “I get sudden feelings of panic”), the HADS-D items pertain to states of anhedonia, the inability to experience pleasure (e.g., “I look forward with enjoyment to things”). Participants were asked how they had been feeling in the past week by responding on a Likert-Type scale ranging from 0 (“not at all”) to 3 (“most of the time”) with higher scores representing higher levels of depression and anxiety. A total score was derived for each subscale by calculating the scores for the seven anxiety items and for the seven depression items.

Past research has provided extensive evidence supporting the reliability and validity of the HADS in a wide range of settings [25]. A systematic review of a large number of studies indicated that the two-factor solution has good internal consistency with Cronbach’s alpha ranging from 0.68 to 0.93 [26].

#### Well-being

The five-item World Health Organisation Well-Being Index (WHO-5) was used to gain a measure of subjective psychological well-being among the fire fighters participating in the study [27]. This short questionnaire consisted of five simple and positively formulated items that reflected the extent to which participants experienced general positive feelings in the last 2 weeks (e.g., “I have felt active and vigorous”). Responses were scored on a six-point Likert-type scale ranging from 0 (“at no time”) to 5 (“all the time”) before being summed. Increasing scores reflected higher levels of wellbeing.

A recent systematic review highlighted the reliability and validity of the WHO-5 both as a screening tool for mental dysfunction and as an outcome measure in clinical trial research [28]. In line with previous research, a recent large-scale study evidenced acceptable internal consistency with a Cronbach coefficient alpha of 0.84 [27]. Furthermore, Topp et al. evaluated the WHO-5 in terms of construct validity and determined that the scale adequately covered the spectrum of the wellbeing construct [28].

### Procedure

The present study was part of a larger randomised controlled trial and ethics approval was obtained from both Monash University (Ref. No.10102) and University of New South Wales (Ref. No. HC15300) to conduct the present research. Fire fighters working within the selected rescue stations received an email from the FRNSW Wellbeing coordinator informing them of the upcoming study. One week later, FRNSW peer support officers visited each station to provide further information on the program and allow fire fighters the opportunity to consider their participation.

Individuals were informed that participation was completely voluntary, and that collected information would remain confidential. Pre-paid envelopes containing consent forms and paper-based questionnaires were distributed to all potential participants. The fire fighters were then asked to complete the questionnaires at a suitable time over the next couple of weeks. Once completed, the fire fighters were instructed to use the reply envelopes to send back the signed consent forms and completed questionnaires to the research team. A total of 238 fire fighters were approached to participate in the study. As outlined above, 143 questionnaires were completed, indicating a response rate of 60%. Unfortunately, no information was available on non-responders.

### Data analysis

Hierarchical regressions were performed using SPSS (v.25; [29] IBM 2013) in order to determine whether levels of mindfulness were associated with anxiety, depression and wellbeing after accounting for age and number of years within FRNSW. Potential confounders, specifically age and years within FRNSW were entered first in the model, while mindfulness was entered subsequently. Proceeding this way provided the capacity to clearly identify the unique contribution of mindfulness in psychological health whilst accounting for the potential confounding impacts of age and experience within FRNSW. Separate models were run for each of the three mental health outcomes to test these hypotheses, using Bonferroni methods to adjust for multiple testing (with Bonferroni adjustment at alpha = .017).

## Results

Data was available for 114 fire fighters. A summary of participant demographics is displayed in Table 1. The vast majority of fire fighters were men but this gender ratio was similar to that found in Australian firefighting organisations at the time of this study.

**Table 1:** Frequency and Percentage of Gender, Age, Years Worked with FRNSW and Education

Measures	Frequency	Percentage
Gender (*N* = 114)		
Male	109	95.6
Female	5	4.4
Age (*N* = 113)		
20–29	9	8.0
30–39	39	34.5
40–49	36	31.8
50–59	29	25.7
Years worked with FRNSW (*N* = 114)		
1–5	11	9.6
6–10	32	28.1
11–15	29	25.4
16–20	9	7.9
Over 20	33	28.9
Education (*N* = 114)		
High School	27	23.7
TAFE	57	50.0
Graduate Degree	27	23.7
Postgrad Degree	3	2.6

*Note. FRNSW* Fire and Rescue New South Wales

Descriptive statistics were inspected for all variables to identify any outliers as well as missing or out-of-range data. While there were no out-of-range data, there were 0.9% missing data for age (*n* = 1) and 1.8% for mindfulness (*n* = 2). An examination of standardised scored data and boxplots indicated that there was one univariate outlier on the anxiety variable. As suggested by Tabachnick and Fidell, the outlier was Winsorised [30].

Results indicated that age and years worked with FRNSW did not statistically predict levels of depression, (*F* (2,108) = .32, *p* = .730), anxiety (*F* (2,108) = .85, *p* = .432) or well-being (*F* (2, 108) = .48, *p* = .623) at the first stage of the model. Nor were these demographic variables significant at stage 2 of the model as can be seen below in Tables 2, 3, and 4. Squared semi-partial correlations for both predictors were close to nil in all models, suggesting that none of these variables contributed to explaining the variability in the mental health dependent variables. Introducing mindfulness, however, significantly improved the prediction of depression (*F* (1,107) = 31.17, *p* ≤ .001), anxiety (*F* (1, 107) = 33.66, *p* ≤ .001) and well-being (*F* (1,107) = 39.62, *p* ≤ .001). Mindfulness accounted for an additional 22.4% of the variability in depression, 23.6% in anxiety and 26.8% in well-being.

**Table 2:** Regression Coefficients and Squared Semi-Partial Correlations for the Hierarchical Multiple Linear Regression Using Age, Years Worked with FRNSW and Mindfulness to Predict Depression

	Variables	*B*	*SE B*	β	95% CI for *B*	*sr* ^*2*^
Step 1						
	Constant	4.58	1.73			
	Age	−.03	.05	−.08	[−.13, .07]	<.01
	Years worked with FRNSW	.26	.33	.11	[−.40, .93]	.01
*R*^*2*^ = .006						
Step 2						
	Constant	12.83	2.12			
	Age	−.02	.05	−.06	[−.11, .07]	<.01
	Years worked with FRNSW	.17	.30	.07	[−.42, .75]	<.01
	Mindfulness	−.23	.04	−.48***	[−.31, −.15]	.22
*R*^2^ Change = .224***; *R*^2^ = .230***						

*Note. B* = unstandardised regression coefficients; SE *B* = standard errors of the unstandardised regression coefficients; β = standardised regression coefficients; CI = confidence interval; *sr*^*2*^ *=* semi-partial correlation squared; FRNSW = Fire and Rescue New South Wales

*** *p* ≤ .001; *N* = 111

**Table 3:** Regression Coefficients and Squared Semi-Partial Correlations for the Hierarchical Multiple Linear Regression Using Age, Years Worked with FRNSW and Mindfulness to Predict Anxiety

	Variables	*B*	*SE B*	β	95% CI for *B*	*sr* ^*2*^
Step 1						
	Constant	3.65	1.91			
	Age	.06	.06	.16	[−.05, .18]	.01
	Years worked with FRNSW	−.15	.37	−.06	[−.88, .58]	<.01
*R*^*2*^ = .015						
Step 2						
	Constant	13.03	2.32			
	Age	.07	.05	.18	[−.03, .17]	.01
	Years worked with FRNSW	−.26	.32	−.10	[−.90, .38]	<.01
	mindfulness	−.26	.04	−.49***	[−.35, −.17]	.24
*R*^2^ Change = .236 ***; *R*^2^ = .251***						

*Note*. *B* = unstandardised regression coefficients; *SE B* = standard errors of the unstandardised regression coefficients; β = standardised regression coefficients; CI = confidence interval; *sr*^*2*^ *=* semi-partial correlation squared; FRNSW = Fire and Rescue New South Wales

*** *p* ≤ .001; *N* = 111

Together, age, years worked at FRNSW, and mindfulness significantly predicted depression (*F* (3,107) = 10.66, *p* ≤ .001), anxiety (*F* (3,107) = 11.95, *p* ≤ .001) and well-being (*F* (3,107) = 13.64, *p* ≤ .001). When all three predictors were included in the model, they explained 23% of the variation in depression (adjusted *R*^*2*^ = .21), while they explained 25.1% of the variability in anxiety (adjusted *R*^*2*^ = .23) and 27.7% of well-being variability (adjusted *R*^*2*^ = .26). Tables 2, 3 and 4 illustrate the regression coefficients together with squared semi-partial correlations for the three dependent variables, depression, anxiety and well-being.

**Table 4:** Regression Coefficients and Squared Semi-Partial Correlations for the Hierarchical Multiple Linear Regression Using Age, Years Worked with FRNSW and Mindfulness to Predict Well-Being

	Variables	*B*	*SE Β*	β	95% CI for *B*	*sr* ^*2*^
Step 1						
	Constant	15.20	2.07			
	Age	.05	.06	.10	[−.08, .17]	<.01
	Years worked with FRNSW	−.40	.40	−.14	[−1.19, .41]	.01
*R*^*2*^ = .009						
Step 2						
	Constant	4.36	2.48			
	Age	.04	.05	.08	[−.07, .14]	<.01
	Years worked with FRNSW	−.26	.35	−.09	[−.95, .42]	<.01
	Mindfulness	.30	.05	.52***	[.20, .39]	.27
*R*^2^ Change = .268 ***; *R*^2^ = .277***						

*Note*. *B* = unstandardised regression coefficients; *SE B* = standard errors of the unstandardised regression coefficients; β = standardised regression coefficients; CI = confidence interval; *sr*^*2*^ *=* semi-partial correlation squared; FRNSW = Fire and Rescue New South Wales

*** *p* ≤ .001; *N* = 111

While age and years worked with FRNSW did not predict any of the outcomes at any stage of the regression, regression coefficients of mindfulness were significant in all models. Fire fighters with higher levels of mindfulness tended to report higher levels of well-being as well as lower levels of depression and anxiety adjusting for age group and the number of years they had worked within FRNSW.

## Discussion

Whereas an extensive body of research has highlighted the positive mental health outcomes associated with mindfulness in the general population [31, 32], little research has been conducted to investigate mental health in the specific context of emergency work [2]. However, mindfulness is considered to be a crucial psychological resource for coping effectively with stress and trauma [15]. Thus, it could potentially represent a modifiable protective factor for the mental health of first responders regularly exposed to chronic and traumatic stressors [20, 21]. In light of these ideas, the aim of the present study was to examine the potential relationships between dispositional mindfulness and mental health in a sample of Australian fire fighters who had been exposed to at least one traumatic incident involving death or serious injury over the last 6 months.

It was specifically hypothesised that higher levels of mindfulness would predict lower levels of anxiety and depression as well as higher levels of psychological well-being, controlling for age and years worked within FRNSW. Results indicated strong evidence in support of this hypothesis. As expected, there was a significant positive association between dispositional mindfulness and well-being coupled with a significant inverse correlation between dispositional mindfulness and reported indices of anxiety and depression. Greater mindfulness was associated with higher levels of psychological well-being as well as lower depression and anxiety symptoms within the sample of fire fighters. In addition, the reported relationships were robust across indicators of mental health with mindfulness explaining a substantial amount of the variability in well-being (26.8%), anxiety (23.6%) and depression (22.4%). However, the demographic variables of age and years worked in FRNSW did not affect the influence of mindfulness on any of the mental health outcomes in our sample.

Present findings replicated previous results linking higher levels of mindfulness to enhanced mental health outcomes, including decreased anxiety, depression [17, 31], general psychopathological symptoms [33], higher psychological well-being [13] and positive affect [32]. The current findings are also consistent with emerging studies conducted with various groups of first responders [20, 34].

Our regression analyses also demonstrated that age and years of service in FRNSW were not predictive for any of the mental health outcomes considered. Neither of these two demographic variables were significantly associated with psychological indices of anxiety, depression or well-being. Such results are partially consistent with the study of Setti and Argentero, where age was not related to mental health but years of fire service was positively correlated with reported post-traumatic symptoms [21]. However, the discrepant pattern of terms of years of fire service is surprising, particularly in light of studies showing a clear link between overall cumulative trauma exposure and a range of mental health outcomes [2]. This result may be partly explained by the fact that the fire fighters involved in the present study were on average almost 5 years older and had more work experience than the Italian fire fighters in the Setti and Argentero study [21]. It may be that length of service, and therefore cumulative trauma exposure, becomes less of a predictor in more experienced first responders, when almost all have had sufficient trauma exposure to precipitate mental distress. Indeed, a recent study determined that any positive correlations between mental disorder and years of service in first responder organisations was only present among staff who were early in their career [8].

The finding that mindfulness benefited the mental health of fire fighters exposed to trauma is congruent with prior research emphasising the importance of self-regulation as a key protective function of mindfulness [13]. Being more aware and accepting of the present moment without judgment may facilitate healthy self-regulation processes as evidenced in correlational research demonstrating the link between higher mindfulness and greater awareness, understanding and attentional capacities as well as increased emotion regulation [13, 35].

In this regard, research has demonstrated that mindfulness could exert its beneficial effects through enhancing self-regulation skills essential to adaptive psychological functioning, including an improved ability to control ruminative thinking [19] as well as negative bias and automatic emotional responses to threat via the insula and amygdala [36]. These findings are in line with prior studies demonstrating that adopting an attitude of acceptance may be beneficial for buffering distress [37]. Indeed, non-reactivity to emotional stimuli could be considered as the operationalisation of acceptance, a crucial dimension of mindfulness reflecting the non-evaluative stance towards the present experience [33]. Adopting an accepting and mindful attitude may foster the capacity to refrain from engaging in impulsive reactions, which has been related to decreased anxiety and depression and increased psychological well-being [17, 37].

Furthermore, present results are consistent with literature suggesting that dispositional mindfulness may be particularly protective for emergency workers who deal with regular trauma exposure [16]. In line with these assumptions, cross-sectional research has previously found that negative relationships between depression and mindfulness were stronger among individuals exposed to high levels of stress compared to those under less stressful conditions [17]. According to Follette et al., mindfulness skills may foster adjustment following traumatic experience through an enhanced processing and integration of trauma-related information as well as a decreased usage of maladaptive avoidant strategies such as withdrawal or substance abuse [16]. While our study was limited to fire fighters who had been exposed to trauma, information about how distressed they felt during the traumatic exposure was not collected. Future research could aim to explore the importance of perceived stress in direct relation to any traumatic event. This could help determine whether the potential protective function of mindfulness in mental health varies according to amount of stress experienced.

Whilst mindfulness, at varying levels, is a trait naturally occurring among humans, research shows that the capacity for mindfulness can be trained with practice [13, 38, 39]. Mindfulness principles have been successfully incorporated in a range of programs aimed at enhancing the psychological well-being of various clinical and non-clinical populations [31, 40]. Current results provide some support for the relevance of considering mindfulness interventions amongst first responders. However, this is an assertion that requires separate testing with intervention studies as it cannot be assumed that trained mindfulness skills will have the same protective effect as dispositional mindfulness.

Although our results suggest that mindfulness could account for about a quarter of the variance of anxiety, depression and well-being amongst first responders, the majority of the variance remains unexplained. Social support has been found to be an important protective factor against the development of psychopathology in adults confronted with traumatic stressors [10]. Consistent with this research, Smith et al. determined that social support was related to reduced depressive symptoms in fire fighters after controlling for mindfulness [20]. Our results are likely to contain unmeasured residual confounding from factors such as social support. Future study may benefit from including social support, as well as other determinants of resilience, in hierarchical regression models to clarify the potential role of mindfulness in the mental health and well-being of first responders.

While this study brings an important contribution to literature on mindfulness and mental health in first responders, there are several limitations. Firstly, the present investigation used self-report questionnaires to assess mental health and mindfulness in fire fighters. Mindfulness may not be fully assessable using self-report due to self-report bias. It may, therefore, be desirable to replicate this study using clinical diagnostic interviews or biomarkers, such as cortisol sampling, to measure psychopathological symptoms of anxiety and depression as well as well-being [40]. The employed scales, however, showed good psychometric qualities and are widely used in research [12, 25, 28, 41]. Secondly, the cross-sectional design of the present study limits the ability to make inferences regarding the direction of causation in the relationship between the observed variables and their underlying constructs. In particular, the possibility remains that some or all of the association between mindfulness and mental health variables is due to the onset of mental health symptoms or poor wellbeing making it more difficult for individuals to utilise mindful techniques. However, research suggests that teaching mindfulness clinically may only be problematic for a minority of trauma survivors with severe symptoms and individuals suffering from severe depression [42]. Thirdly, there may be a problem of multicollinearity in some of the models presented, most notably between age and years of service within FRNSW, which were found to be strongly correlated (*r* = .74). However, Field [43] has suggested that only correlation values greater than .80 could jeopardise the validity of the regression model estimates. In order to confirm that the level of correlation between these two variables was not influencing our results, sensitivity analyses were conducted, in which each of the regression models were reconstructed, but only controlling for years worked with FRNSW. As demonstrated in Additional file 1, the overall conclusion remained unchanged. Finally, the fire fighters involved in the study may not be thoroughly representative of fire fighters located in other geographical regions. In particular, our sample had an overwhelming majority of males, meaning we were not able to explore gender differences with any statistical power and remain unsure if these findings are relevant for female workers. Findings would need to be replicated in various emergency groups in different environments and contexts.

## Conclusions

The present study provides evidence for robust positive associations between dispositional mindfulness and psychological health in Australian fire fighters exposed to trauma. While further investigations are required to substantiate these findings, this research has important implications. Mindfulness is a psychological characteristic that may be able to be modified, although further research is required to test whether taught mindfulness has the same positive benefits as demonstrated with dispositional mindfulness.

## Additional file

Additional file 1: **Table S1.** Regression Coefficients and Squared Semi-Partial Correlations for the Hierarchical Multiple Linear Regression Using Years Worked with FRNSW and Mindfulness to Predict Depression. **Table S2.** Regression Coefficients and Squared Semi-Partial Correlations for the Hierarchical Multiple Linear Regression Using Years Worked with FRNSW and Mindfulness to Predict Anxiety. **Table S3.** Regression Coefficients and Squared Semi-Partial Correlations for the Hierarchical Multiple Linear Regression Using Years Worked with FRNSW and Mindfulness to Predict Well-Being. (DOCX 23 kb)

## Data Availability

The datasets used and/or analysed during the current study are stored on the UNSW repository and are available from the corresponding author on reasonable request. Because of the sensitive nature of the data collected on the mental health of a group of workers amongst which individuals are potentially identifiable, we cannot provide open access to our data. External access to research data will be subject to approval by UNSW Human Research Ethics Committee.

## Abbreviations

*B*: Unstandardised regression coefficients
CI: Confidence interval
FMI-14: Freiburg Mindfulness Inventory
FRNSW: Fire and Rescue New South Wales
HADS: Hospital Anxiety and Depression Scale
HADS-A: Hospital Anxiety and Depression Scale, subscales for anxiety
HADS-D: Hospital Anxiety and Depression Scale, subscales for depression
PTSD: Post-traumatic stress disorder
*SE B*: Standard errors of the unstandardised regression coefficients
*sr*^*2*^: Semi-partial correlation squared
WHO-5: Five-item World Health Organisation Well-Being Index
β: Standardised regression coefficients
