# Supplementary material for: Mental health and mindfulness amongst Australian fire fighters
Source: BMC Psychol. 2019 Jun 14;7:34. doi: 10.1186/s40359-019-0311-2 (PMC6570940; doi:10.1186/s40359-019-0311-2)
Supplement: Supplementary file 1 — Table S1. Regression Coefficients and Squared Semi-Partial Correlations for the Hierarchical Multiple Linear Regression Using Years Worked with FRNSW and Mindfulness to Predict Depression. Table S2. Regression Coefficients and Squared Semi-Partial Correlations for the Hierarchical Multiple Linear Regression Using Years Worked with FRNSW and Mindfulness to Predict Anxiety. Table S3. Regression Coefficients and Squared Semi-Partial Correlations for the Hierarchical Multiple Linear Regression Using Years Worked with FRNSW and Mindfulness to Predict Well-Being. (DOCX 23 kb) [file 40359_2019_311_MOESM1_ESM.docx]

Table S1

*Regression Coefficients and Squared Semi-Partial Correlations for the Hierarchical Multiple Linear Regression Using Years Worked with FRNSW and Mindfulness to Predict Depression*

|  | Variables | *B* | *SE B* | β | 95% CI for *B* | *sr^2^* |
| --- | --- | --- | --- | --- | --- | --- |
| Step 1 |  |  |  |  |  |  |
|  | Constant | 3.72 | .57 |  |  |  |
|  | Years worked with FRNSW | .12 | .22 | .05 | [-.32, .56] | <.01 |
| *R^2^* = .003 | | | | | | |
| Step 2 |  |  |  |  |  |  |
|  | Constant | 12.21 | 1.58 |  |  |  |
|  | Years worked with FRNSW | .06 | .19 | .03 | [-.33, .45] | <.01 |
|  | Mindfulness | -.23 | .04 | -.48*** | [-.31, -.15] | .23 |
| *R*^2^ Change= .226***; *R^2^*= .229*** | | | | | | |
| *Note. B* = unstandardised regression coefficients; SE *B* = standard errors of the unstandardised regression coefficients; β = standardised regression coefficients; CI = confidence interval; *sr^2^=* semi-partial correlation squared; FRNSW = Fire and Rescue New South Wales.  **p* ≤ .05, ***p*<.001, *** *p* ≤ .001; *N* = 111 | | | | | | |

Table S2

*Regression Coefficients and Squared Semi-Partial Correlations for the Hierarchical Multiple Linear Regression Using Years Worked with FRNSW and Mindfulness to Predict Anxiety*

|  | Variables | *B* | *SE B* | β | 95% CI for *B* | *sr^2^* |
| --- | --- | --- | --- | --- | --- | --- |
| Step 1 |  |  |  |  |  |  |
|  | Constant | 5.76 | .64 |  |  |  |
|  | Years worked with FRNSW | .14 | .25 | .05 | [-.35, .62] | <.01 |
| *R^2^* = .003 | | | | | | |
| Step 2 |  |  |  |  |  |  |
|  | Constant | 15.37 | 1.76 |  |  |  |
|  | Years worked with FRNSW | -.07 | .22 | .03 | [-.36, .49] | <.01 |
|  | mindfulness | -.26 | .05 | -.48*** | [-.35, -.17] | .23 |
| *R^2^* Change = .233 ***; *R^2^* = .236*** | | | | | | |
| *Note*. *B* = unstandardised regression coefficients; *SE B* = standard errors of the unstandardised regression coefficients; β = standardised regression coefficients; CI = confidence interval; *sr^2^=* semi-partial correlation squared; FRNSW = Fire and Rescue New South Wales  **p* ≤ .05, ***p* <.001, *** *p* ≤ .001; *N* = 111 | | | | | | |

Table S3

*Regression Coefficients and Squared Semi-Partial Correlations for the Hierarchical Multiple Linear Regression Using Years Worked with FRNSW and Mindfulness to Predict Well-Being*

|  | Variables | *B* | *SE Β* | β | 95% CI for *B* | *sr^2^* |
| --- | --- | --- | --- | --- | --- | --- |
| Step 1 |  |  |  |  |  |  |
|  | Constant | 16.54 | .69 |  |  |  |
|  | Years worked with FRNSW | -.16 | .27 | -.06 | [-.68, .37] | <.01 |
| *R^2^* = .003 | | | | | | |
| Step 2 |  |  |  |  |  |  |
|  | Constant | 5.34 | 1.86 |  |  |  |
|  | Years worked with FRNSW | -.07 | .23 | -.03 | [-.52, .38] | <.01 |
|  | Mindfulness | .30 | .05 | .52*** | [.21, .39] | .27 |
| *R^2^* Change= .270 ***; *R^2^* = .273*** | | | | | | |
| *Note*. *B* = unstandardised regression coefficients; *SE B* = standard errors of the unstandardised regression coefficients; β = standardised regression coefficients; CI = confidence interval; *sr^2^=* semi-partial correlation squared; FRNSW = Fire and Rescue New South Wales.  **p* ≤ .05, ***p* <.001, *** *p* ≤ .001; *N* = 111 | | | | | | |
